# Investigating the Effects of Skills-Based Training Interventions on Executive and Psychosocial Functioning in Adults with ADHD – A Systematic Review of Practical, Emotional and Cognitive Approaches

**DOI:** 10.1192/bjo.2026.11237

**Published:** 2026-06-30

**Authors:** Caitlin Evans, Jac Airdrie, Sian Edney

**Affiliations:** Cardiff University, Cardiff, United Kingdom

## Abstract

**Aims:**

Executive dysfunction is central to the core symptoms of Attention Deficit Hyperactivity Disorder (ADHD) and reflects complex, goal-directed behaviours linked to psychosocial and cognitive outcomes. There is a gap in understanding the impact of practical, skills-based interventions on executive functioning in adults with ADHD. This review aimed to evaluate the effectiveness of skills-based interventions on executive functioning, with a secondary focus on psychosocial outcomes.

**Methods:**

The review was conducted according to PRISMA guidelines. Searches were conducted on 4 July 2025 across PsychINFO, Medline, Web of Science, Embase and Scopus for studies published from 2013. Peer-reviewed studies examining the effects of skills-based training interventions on executive and psychosocial outcomes in adults with ADHD were eligible. The methodological quality of included studies was assessed for risk of bias and certainty of evidence (GRADE).

**Results:**

The search yielded 12 studies testing the efficacy of interventions,grouped into four categories: goal-orientated interventions (k=4), occupation-focused skills training (k=2), cognitive training with executive coaching (k=2), and Emotion regulation interventions (k=4). Goal-orientated interventions showed mixed results. Only one controlled study found limited between-group improvements, while the others showed none. Non-controlled studies reported several within-group gains, at follow-up but without control groups, improvements cannot be attributed to the intervention alone. Psychosocial outcomes varied, with two controlled studies showing improvements compared to controls, while the remaining studies showed only within-group change. Non-controlled studies showed within-group improvements across psychosocial outcomes, with effects in emotion-regulation interventions, but without control groups, these gains cannot be attributed to interventions. Occupation-focused skills training demonstrated the most consistent within-group improvements, with both studies reporting significant pre-post gains across executive fucntioning domains and quality of life. Cognitive training and executive coaching produced improvements invisuo-spatial working memory, but little evidence of change in daily functioning. Emotion-regulation interventions showed stronger effects, with improvements in executive functioning and moderate-to-large effects compared with self-guided controls, but psychosocial outcomes were less consistently improved.

**Conclusion:**

Skills-based interventions show potential for improving executive functioning and, to a lesser extent, psychosocial outcomes in adults with ADHD. Occupational-focused and emotion-regulation based interventions demonstrated the most consistent benefits while cognitive and goal-orientated interventions showed mixed results. Small samples, heterogeneity in intervention type, study design and outcome measures restrict conclusions. Future research should prioritise robust controlled designs, larger and more diverse samples, and direct comparisons of different skills-based approaches to clarify which interventions yield the greatest impact.

